# Sudan's mental health service: challenges and future horizons

**DOI:** 10.1192/bji.2019.19

**Published:** 2020-02

**Authors:** Abdelgadir H. M. Osman, Aisha Bakhiet, Samia Elmusharaf, Abdelaziz Omer, Abdalla Abdelrahman

**Affiliations:** 1 FRCPsych, Associate Professor, Department of Psychiatry, Faculty of Medicine, PO Box 102, University of Khartoum, Khartoum, Sudan. Email: abdelgadir1159@yahoo.com; 2 Senior Lecturer, Department of Psychiatry, Faculty of Medicine, University of Khartoum, Sudan

**Keywords:** Transcultural psychiatry, low- and middle-income countries, history of psychiatry

Sudan is located to the south of Egypt, along the Red Sea. The country has a total area of 1 886 068 km^2^ (728 215 square miles) and a population of just over 37 million and is classified by the World Bank as a low-income country. The adult literacy rate is 59%. The total annual expenditure on healthcare is 4.3% of the gross domestic product, but the proportion spent on mental healthcare is unknown. As of 2009, there were just 0.09 psychiatrists and 0.2 psychiatric nurses per 100 000 population, and 0.2 mental health beds per 10 000 people, of which 90% were hospital-based; the other 10% were community-based units run by physician assistants and psychologists.^1,2^

## Mental health policy

Sudan's mental health policy, which was last published in 2008, proposes the following components: developing a mental health component in primary healthcare, scaling up human resources, involvement of patients and their families, strengthening advocacy, promotion of the human rights protection of patients, equity and access to mental healthcare services across different groups, quality improvement, financing and monitoring systems. The publication of this national policy and strategy was followed by another document in 2009, proposing restructuring the mental healthcare system in the country. The drafting of these documents attracted considerable participation by leading psychiatrists working in Sudan, as well as technical support from the World Health Organization (WHO) regional office.^3,^^4^

## Legislation and the Mental Health Act

Although a Mental Health Act was drafted in 1998, Parliament approved it only in June 2018. The Act features the following components: access to mental healthcare, including access to the least restrictive care; the rights of mental health service patients, their families and other caregivers; competency, capacity and guardianship issues for people with mental illness; voluntary and involuntary treatment; appeal and review systems; law enforcement and other judicial system issues for people with mental illness.^5^

## Mental health set-up and workforce

Although mental healthcare services in Sudan are theoretically free for patients, only those living in urban areas have access to out-patient clinics and can therefore be admitted to a psychiatric unit if required. Most psychiatric services are concentrated in Khartoum, the capital city, where there are six psychiatric hospitals, two of which provide specialised services for forensic patients. Another is for drug and alcohol rehabilitation, and is run by a quasi-governmental body, requiring fees for the use of its service. Out of the 18 federal states, only 12 have fully equipped psychiatric hospitals run by qualified consultant psychiatrists. The other six are either managed by non-specialist medical doctors or by clinical psychologists and medical assistants.

Table 1 gives updated figures from the Ministry of Health. The total number of mental health professionals working in all sectors, including the private sector and services run by non-governmental organisations (NGOs), is 899. Of these, 554 (61.5%) work in formally constituted mental health units and hospitals; 350 of these individuals are doctors who are either in psychiatric training schemes or non-specialist doctors who run primary care centres and associated units. Young doctors often move jobs to further their careers and ambitions, and psychiatrists in training may not stay in the country. The workforce figures hide massive regional inequities; only a small proportion of psychiatrists work in rural areas, which are home to two-thirds of Sudan's population.

**Table 1:** Mental health professionals in Sudan^2^^,^^17^

Profession	All in private and public sector	Public sector	NGOs	Private sector	Per 100 000
Psychiatrists	81	48	2	31	0.21
Psychologists	262	65	37	160	0.69
Social workers	38	38	NK	NK	0.16
Doctors in training	97	95	2	NK	0.20
Trained medical practitioners	345	255	3	1	0.91
Psychiatric nurses	76	71	0	5	0.2
Mental health staff only	554	317	7	2	1.6

NGOs, non-governmental organisations; NK, data not known.

## Prevalence of psychiatric disorders

Although no national prevalence study has been conducted across the whole country, many articles have been published addressing the psychiatric needs of specific sectors or groups. These include schoolchildren, perinatal care, internally displaced persons, out-patients and community catchment areas. The following are indicative examples of findings from published prevalence studies. The prevalence of depression and anxiety in high-school students in Khartoum State has been estimated to be 12%.^6^ The prevalence of perinatal psychiatric disorders in primary care settings and communities in the capital city of Sudan has been estimated at 23%.^7^ Higher rates of psychiatric disorders have been found among internally displaced persons (53%),^8^ including major depressive disorder (24.3%), generalised anxiety disorder (23.6%), social phobia (14.2%) and post-traumatic stress disorder (12.3%). The prevalence rate for major psychotic disorders among the internally displaced population is 1.5%;^9–11^ no data are available for suicide attempts, completed suicides, or drug and alcohol addiction.

## Mental health service provision

Access to mental health facilities is unevenly distributed across the country, being more available to those living in or near the capital city and, as emphasised, there are severe shortages of trained personnel. Many communities throughout Sudan use traditional and religious healers to help meet their primary healthcare needs. Besides being accessible and available, traditional healers are often part of the wider cultural belief system and are considered integral to everyday life and well-being. Attempts have been made to promote a dialogue between mental health professionals and traditional healers. As a consequence, there are many traditional healing centres that embrace integral working models of modern psychiatry and receive regular input from medical assistants, who have participated in special training in modern psychiatry, to provide diagnoses and intervention.^12,13^

## Education and psychiatric training

Out of the 32 medical schools in Sudan, 17 incorporate full psychiatric and behavioural science into their curriculum, including a clinical skills-based component. Twenty-two teach psychiatry as a separate course and discipline, while the other ten teach it as part of general medicine. Sudan trains over 3000 new doctors each year, but most will emigrate within a few years.^14^

Postgraduate mental health training in Sudan started in 1990 under the oversight of the Faculty of Medicine at the University of Khartoum (all postgraduate training is now overseen by the Sudan Medical Specialization Board). A clinical MD is offered, in a 4-year programme comprising examinations and a research-based thesis. So far, 178 graduates have qualified to become specialists in psychiatry, 75% of whom have since emigrated, mostly to work in the Gulf States. In response, the Ministry of Health, together with the Gezira University health programme, with substantial support from the WHO local office, developed a novel scheme to support psychiatry guided by the WHO's Mental Health Gap Action Programme (mhGAP)^15^ Intervention Guide.^16^ This scheme aimed to train primary care physicians in the diagnosis and treatment of common psychiatric disorders. Over 400 primary care doctors have passed through the scheme to date.

## Scaling up mental health service in Sudan

Researchers, policymakers and international agencies have issued calls for low- and middle- income countries to scale up the mental health components of their healthcare systems by capacity building in the workforce.^2,^^17^ The WHO mhGap document^15^ recommended that Sudan needs least six mental healthcare workers per 100 000 population. To accomplish this, Sudan needs to greatly increase its mental health workforce, from the current figure of just 1.6 per 100 000 population, over the next decade. To achieve this, a new model of service delivery should be developed that delivers sustainable services. This could involve training lay counsellors, psychologists, nurses and primary care doctors to provide early interventions at the grass roots, in urban and especially rural populations. Academic departments and research units, together with the policy-making department at the Ministry of Health in Sudan, should spearhead such changes, in collaboration with regional and international organisations.
